# Cryopreservation of human cortical organoids using vitrification

**DOI:** 10.3389/fnmol.2026.1913908

**Published:** 2026-09-08

**Authors:** Kyle Stokes, Sandra Mojica-Perez, Samantha Jacobs, Joy Huang, Shivanshi Vaid, Yukun Yuan, Caroline A. Pearson, Daniel Montes, Andrew Tidball, Debora VanHeyningen, Jonathan Gaillard, Louis T. Dang, Margaret Elizabeth Ross, Lori L. Isom, Michael Uhler, Wei Niu, Jack M. Parent

**Affiliations:** 1 Department of Neurology, University of Michigan, Ann Arbor, MI, United States; 2 The Human Stem Cell and Gene Editing Core, University of Michigan, Ann Arbor, MI, United States; 3 Department of Pediatrics, University of Michigan, Ann Arbor, MI, United States; 4 Department of Pharmacology, University of Michigan, Ann Arbor, MI, United States; 5 Laboratory of Neurogenetics and Development, Brain and Mind Research Institute, Weill Cornell Medical College, New York, NY, United States; 6 Michigan Neuroscience Institute, University of Michigan, Ann Arbor, MI, United States; 7 Department of Molecular and Integrative Physiology, University of Michigan, Ann Arbor, MI, United States; 8 Department of Biological Sciences, University of Toledo, Toledo, OH, United States; 9 VA Ann Arbor Healthcare System, Ann Arbor, MI, United States

**Keywords:** brain organoid, cerebral organoid, human *in vitro* model, human pluripotent stem cells, neurodevelopment

## Abstract

Vitrification is a fast-cooling cryopreservation technique that limits ice crystal formation and cryoprotectant toxicity. We adapted an established vitrification method used on oocytes and blastocysts and optimized it for the cryopreservation of single and multi-rosette cortical organoids. Vitrified and rewarmed organoids were compared to unvitrified controls using immunofluorescence, qRT-PCR, and electrophysiological recordings. Vitrified and rewarmed organoids generated using two different protocols showed similar levels of proliferating cells, cell death and cell-type specific markers as unvitrified controls. We also found via multielectrode array and patch-clamp recordings that the vitrified and rewarmed organoids showed similar activity to unvitrified controls. Preliminary evidence suggests that vitrified cortical organoids could be stored for over a year and shipped long distance with little detriment to the hCOs after rewarming. Our vitrification cryopreservation method allows for the long-term storage of hCOs at ultralow temperatures and rewarming with minimal impact on cell-type specification or electrophysiology. This method provides a useful alternative approach for bio-banking and cross-institutional collaboration using cortical organoids as a model system.

## 1 Introduction

Human brain organoid models are self-organizing three-dimensional (3-D) culture systems that recapitulate many aspects of brain development and function. They have become an invaluable tool for understanding human biology. These culture systems have a higher cellular diversity than two-dimensional (2-D) cultures and allow for more complex cell–cell interactions (Clevers, 2016; Kim et al., 2020). Organoid systems have been widely used to study organ/tissue development, disease modeling, and to evaluate therapeutic efficacy. Although not all have been formally… quantified, several obstacles are known to exist in organoid research, including the cost of reagents, time to full maturation, untimely experimental termination, and batch-to-batch variation (Zhao and Haddad, 2024). The ability to cryopreserve and then reconstitute organoids would enable investigators to preserve or share organoids from the same batch to mitigate these barriers.

Conventional cryopreservation methods utilize a freezing medium containing 5–10% Dimethyl-Sulfoxide (DMSO) and slow cooling (1–2 °C/min) in a − 80 °C freezer overnight (Li et al., 2010). Cryogenic vials are then transferred to liquid nitrogen (LN_2_) at −195 °C for long-term storage. This slow-cooling cryopreservation approach works well for several organoid systems, including gastrointestinal (Prinelli et al., 2021), lung (Demchenko et al., 2023), liver (Kim et al., 2022), and retina (Reichman et al., 2017). However, this method is less suitable for cortical organoids and human brain tissue due to their fragile architecture. Two cryopreservation methods were recently published demonstrating the ability to cryopreserve brain organoids using slow-cooling methods (Ramani et al., 2024; Xue et al., 2024). One protocol used a novel medium with improved cryoprotective ability composed of methylcellulose, ethylene glycol, DMSO and the Rho Kinase inhibitor Y-27632 (MEDY) while the other used a commercially available cryopreservation solution (CS10, Stemcell Tech.). However, each protocol has drawbacks limiting utility.

Cryoprotectants (CPAs), including the ones listed above, are used to limit ice crystal formation. Due to the high water content in the cryopreservation medium, ice crystal formation is inevitable using the slow freezing approach (Mazur, 1984). Both the formation of ice crystals during freezing and ice melting during thawing disrupt intra- and extra-cellular structures, resulting in cell damage or death (Taylor and Pegg, 1983; Karlsson and Toner, 1996; Pegg, 2010). Adjusting concentrations of CPAs together with rapid warming is sufficient to prevent significant cell death in some organoid models (Reichman et al., 2017; Liu et al., 2021; Prinelli et al., 2021; Kim et al., 2022; Demchenko et al., 2023). To avoid the risks associated with the slow freezing method, we opted to utilize a vitrification strategy which has been shown to work well on very sensitive cell types like oocytes as well as whole embryos (Kuwayama, 2007; Nagy et al., 2020).

The vitrification (“turn to glass”) approach to cryopreservation is the process of turning a liquid phase material into a solid phase through a sudden increase of viscosity which limits ice crystallization (Fahy et al., 1984). The vitrification method of cryopreservation is most frequently used to cryopreserve oocytes and embryos for *in vitro* fertilization (IVF) (Kuwayama, 2007; Nagy et al., 2020). Vitrification entails exposing cells or tissue to high concentrations of CPAs followed by a rapid surface cooling rate of blastocysts (~2–5 × 10^6^ °C/min) (Kuwayama, 2007; Nagy et al., 2020), compared to the conventional method that cools at approximately 1–2 °C/min (Fahy and Wowk, 2015). Fast cooling causes the liquid in cells or tissues to solidify, thereby limiting crystallization. Surface warming after vitrification is also rapid (~3 × 10^3^ °C/min) to prevent ice formation followed by several washing steps to avoid CPA toxicity and osmolarity shock (Kuwayama, 2007; Nagy et al., 2020). To date, vitrification has been used on five 3D cell culture systems: intestinal (Spurrier et al., 2014), lung cancer (Liu et al., 2021), testicular (Pendergraft et al., 2017), mesenchymal stem cell spheroids (Jeong et al., 2020), and neuroectoderm spheroids (Eiraku et al., 2008) but has not yet been shown to work on brain organoids. An elaborate vitrification and rewarming protocol was recently applied to whole rat kidneys, and liver demonstrating that vitrification can preserve complex organ function as assessed after transplantation (Han et al., 2023; Sharma et al., 2023). Taken together, the above work indicates that vitrification is an effective method for preserving tissues with complex structures.

Since slow cooling methods in our hands had been unsatisfactory for human cortical organoids (hCOs) and we had previous experience with vitrification, we adapted a vitrification protocol used to preserve oocytes and blastocysts in this study. Here, we describe a vitrification approach that allows for the cryopreservation and rewarming of hCOs. We also offer preliminary evidence of long-term storage capabilities and find that hCOs preserved using this approach develop similarly to non-cryopreserved organoids and can be shipped between institutions.

## 2 Materials and methods

### 2.1 hPSC culture

All hPSC experiments were conducted following prior approval from the University of Michigan Human Pluripotent Stem Cell Research Oversight (HPSCRO) Committee. Male A2 (Pan et al., 2025) and CON2E (Tidball et al., 2017) lines were reprogrammed to induced pluripotent stem cells (iPSCs) from human foreskin fibroblasts using a previously described protocol (Tidball et al., 2017). The female 79B iPSCs were reprogrammed from the blood of a healthy female that we purchased from ZenBio (#SER-WB10ML-SDS) (Supplementary Figure 1). The P19 (Tidball et al., 2023), 506, and 5ll cell lines were derived from the female WA09 (H9) human embryonic stem cell (hESC) line (WiCell, # WB68074) and subjected to CRISPR gene editing. The P19 and 506 lines were unedited despite exposure to the CRIPSR reagents and used as isogenic controls in published (Tidball et al., 2023) and unpublished work. The 511 line is a CRISPR edited line with a heterozygous mutation in *STXPB1* generated by using the Precision gRNA synthesis kit (Thermo, #A29377) and transfected with TrueCut™ Cas9 protein v2 (Thermo, #A36496) using the Neon™ transfection electroporation system (Thermo, #NEON1S). The sgRNA sequence is CGTAGACAAACTCTGCCGAG targeting *STXBP1*. Single nucleotide polymorphism DNA microarray (SNP-Chip) was performed to confirm genomic integrity.

hPSC lines were maintained in feeder-free conditions and cultured on Geltrex-coated (1:100 dilution; Thermo, #A1413302) 6-well plates (Thermo, #11330032) in either mTeSR1, mTeSR™, Plus (STEMCELL Tech, #85850; #100–0276) or StemFlex™, (Gibco, #A3349401) stem cell maintenance medium at 37 °C with controlled humidity and 5% CO_2_. Cells were routinely passaged at 80% confluency by washing with Dulbecco’s phosphate buffered saline without calcium and magnesium (DPBS; Gibco, #14190144), then lifted off the plate by incubating in 0.8 mM ethylenediaminetetraacetic (EDTA) for 5 min diluted from AccuGENE^®^ 0.5 M EDTA Solution (Lonza, #51201) (Mojica-Perez et al., 2023). Mycoplasma testing was carried out periodically using PCR amplification of ribosomal DNA of mycoplasma with the primer set (5′-TGCACCATCTGTCATTCTGTT-3′ and 5′-GGGAGCAAACAGGATTAGATA-3′).

### 2.2 Organoid differentiation protocols

#### 2.2.1 SOSR-CO differentiation

The self-organizing single rosette organoid (SOSR-CO) protocol was modified slightly from our published method (Tidball et al., 2023). Briefly, hPSC lines between passage 8–35 were passaged using StemPro™ Accutase™ (Thermo, #A1110501) and replated onto Geltrex-coated (1,50 dilution in DMEM/F12), 12-well plates at 6 × 10^5^–2 × 10^6^ cells/well (cell line dependent) in either mTeSR™, Plus or StemFlex™, with 10 μM rho-kinase inhibitor (Y-27632; Tocris, #1254). Rho-Kinase inhibitor was removed the next day, and 2 mL of stem cell medium was replaced daily until cells reached 80–100% confluency. A full media change was done with modified 3N culture medium (Shi et al., 2012) without vitamin A, supplemented with 3 inhibitors to facilitate differentiation down a neuronal path: 2 μM DMH1 (Tocris, #4126), 2 μM XAV939 (Cayman Chemical, #13596), and 10 μM SB-431542 (Cayman Chemical, #13031) (Day 0). Daily 75% media changes with the addition of 1 μM cyclopamine (Cayman Chemical, #11321) were performed on days 1–3. On day 4, the monolayer was cut into squares using the StemPro EZ passage tool (Fisher, #23181010), liberated using a hypertonic sodium citrate solution (Nie et al., 2014) and washed with preconditioned 3 N medium. Liberated squares were diluted with an additional 2 mL of fresh 3 N culture medium containing 4 inhibitors (DMH1, XAV939, SB-431542, Cyclopamine). A total of 200 μL of resuspended squares were then transferred into each well of a 96-well plate preincubated at 37 °C for 30 min with 35 μL of 100% Geltrex/well. On day 6, a half medium change was performed with 3 N media containing 3 μM CHIR99021 (Cayman, #13122). On Day 10 the organoids were removed using the STRIPPER^®^ Micropipetter with a bent 275 μM tip (Cooper Surgical, #MXL3-STR; #MXL3-275) and plated individually in a low adherence U-bottom 96-well plate (Corning, #7007) with 200 μL of 3 N medium without vitamin A and with 3 μM CHIR99021, 20 ng/mL Brain Derived Neurotrophic Factor (BDNF) and 20 ng/mL Neurotrophic factor-3 (NT3) (Peprotech, #450–02; #450–02). On day 12, half-media changes every other day were initiated with 3 N with vitamin A, 20 ng/mL BDNF and 20 ng/mL NT3. From day 30 onward SOSR-COs were transferred to low-adherence 24-well plates (Corning, #3475) in 400 μL 3 N media with vitamin *A. media* was exchanged every other day after day 30.

#### 2.2.2 Multi-rosette-hCO differentiation

This protocol was modified slightly from the previously published version (Paşca et al., 2015). Briefly, hPSCs were dissociated to single cells using Accutase, and 3 × 10^6^ cells were added to a single well of an Aggrewell 800 plate (STEMCELL Tech, #34811) with 2 mL of mTesR1 medium (STEMCELL Tech, #85850) containing 50 μM Rho-Kinase inhibitor (Day−1). A full media change was performed the following day (Day 0) by replacing mTesR1 with Neural Induction Media [NIM; Neurobasal-A (Thermo, #A2477501), 20% Knockout serum replacement (Thermo, 10828028), 0.5% GlutaMAX 100X (Thermo, #35050061), 1% MEM NEAA (Thermo, #11140035), 1% Pen/Strep (Thermo, 15140122), 10 μM β-mercaptoethanol (Thermo, 21985023)] containing 5 μM Dorsomorphin (Tocris, #3093) and 10 μM SB-431542. On day 1, embryoid bodies (EBs) were formed and transferred to a 100 mm dish (Corning, #430591) with 10 mL NIM medium containing Dorsomorphin and SB-431542 and placed on a rotating shaker at 37 °C with 5% CO_2_. Daily media changes were done until day 5. From days 6–14, media was exchanged daily with Neural Media [NM; Neurobasal-A, 1% GlutaMAX, 1% Pen/Strep, 2% B-27 Supplement -A (Thermo, #12587010)] containing 20 ng/mL Epidermal Growth Factor (EGF; Millipore, #01–102) and 20 ng/mL Fibroblast Growth Factor-2 (FGF2; R&D Biosystems, #233-FB). On day 15, organoids were removed from the shaker and transferred into a 6-well plate (10/well) to avoid overcrowding, and the media was exchanged every other day. On day 25, the media was switched to NM containing 20 ng/μL BDNF and 20 ng/μL NT3 and changed every other day. From day 43 onward the media was exchanged every fourth day with NM only.

### 2.3 Cryopreservation and rewarming protocol

#### 2.3.1 Vitrification and rewarming

**Figure 1:**
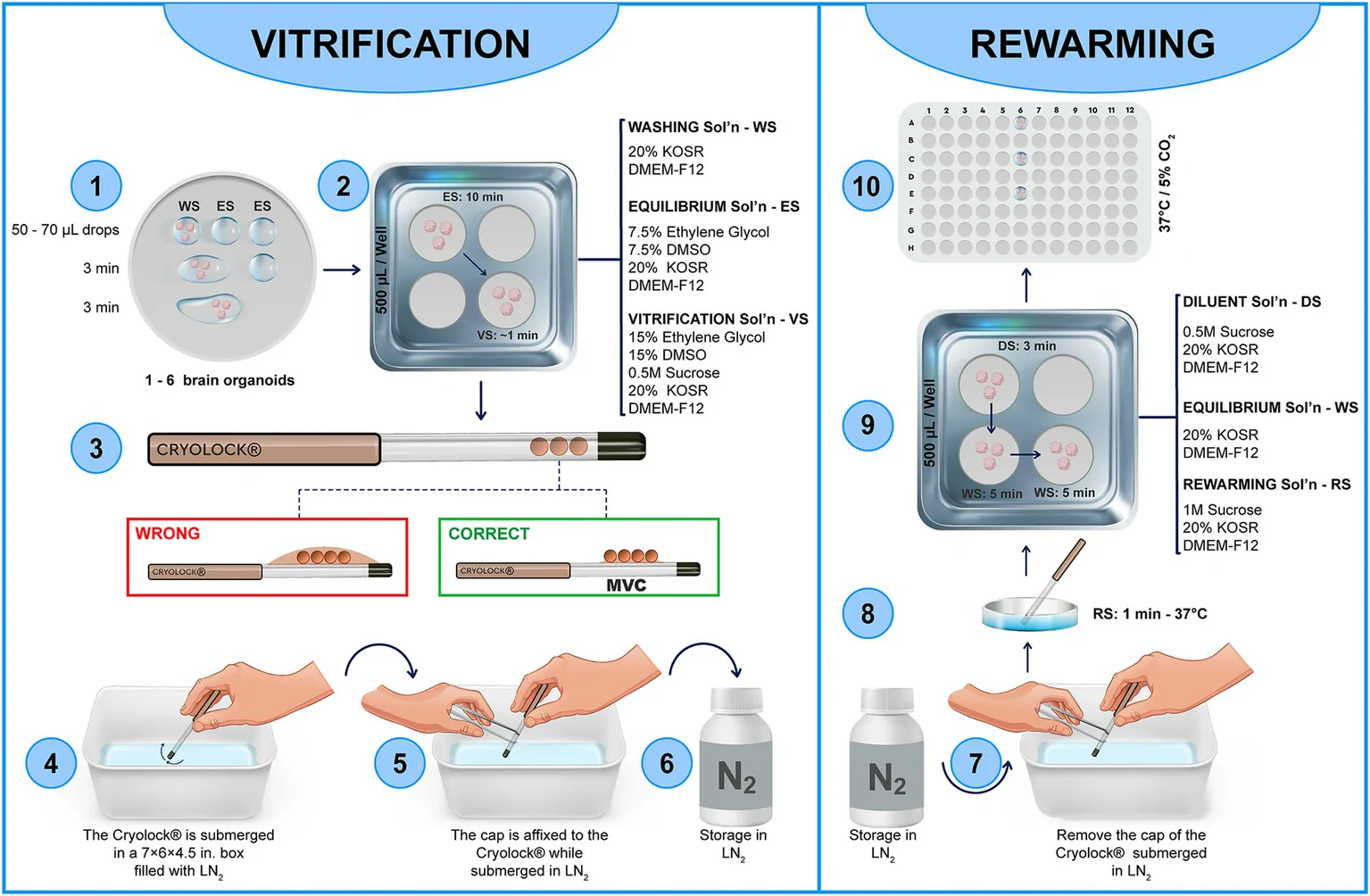
Overview of the vitrification protocol. Left: Human cortical organoids (hCOs) are vitrified by sequential placement in a series of solutions containing increasing levels of cryoprotectants (CPAs) (steps 1 and 2). hCOs are then placed in a Cryolock^®^ and all the CPA is removed (3) followed by immediate immersion in LN_2_ for minimum volume cooling (MVC; 4–5). These vitrified hCOs are then transferred to a cryo-storage tank for long-term storage (6). Right: Rewarming of hCOs requires immediate immersion of hCOs into a 37 °C rewarming solution (steps 7 and 8) followed by sequential removal of CPAs (9 and 10).

In a HERA Guard clean bench (Thermo) using a Stripper^®^ CC micropipetter with 600 μm tip (Cooper Surgical, #MXL3-STR-CC; #MXL3-600), 3–6 (size dependent) day 20 pd and 35 pd hCOs were placed into a 70 μL droplet of washing solution (WS) which was then merged with a droplet of equilibration solution (ES) and incubated for 3 min at room temperature (RT). A second bead of ES was merged with the first two and incubated for another 3 min. hCOs were then transferred to 1 well of a 4-well plate (Fisher, #14444) containing 500 μL ES and incubated for 10 min at RT. hCOs were again transferred to a well continuing 500 μL vitrification solution (VS) for 1 min then immediately transferred to a Cryolock^®^ (IVFStore, #SCL-R-CT-B) where all remaining media was removed. The Cryolock^®^ (blastocyst surface cooling rate of −19,800 °C/Min in LN_2_) was then submerged into liquid nitrogen (LN_2_) and capped while still submerged. The capped Cryolock^®^ containing the hCOs was then transferred to a 13 mm Goblet affixed to a Cryo-Cane (IVF store, #13-100-CLR, Thermo, #5015–0002) and placed in LN_2_ tank for long-term storage. For rewarming, the Cryolock^®^ was rapidly transferred from the LN_2_ dewar to a polystyrene container filled with LN_2_, the Cryolock^®^ was then uncapped while submerged followed by immediate submersion in 2 mL 37 °C rewarming solution (RS) in a 35 mm petri dish (Greiner, #627161) for 1 min (blastocyst surface warming rate of +27,000 °C/Min in LN_2_). hCOs were transferred using a cut-off p200 pipette to a well containing 500 μL diluent solution (DS) and incubated for 3 min at RT. We then performed 2 × 5-min incubations in 500 μL WS at RT. hCOs were then transferred into native media for continued culture. See Table 1 for solution compositions. Detailed protocol descriptions can be found in Figure 1 and Supplementary Videos 1, 2, as well as Supplementary experimental procedures.

**Table 1:** Vitrification solutions.

Reagent	Manufacturer	Cat. #	Concentration based on a 10 mL volume	
Washing solution (WS)				
Knockout serum replacement (KOSR)	Invitrogen	#10828010	20%	2 mL
DMEM F/12 (15 mM HEPES)	Invitrogen	11330032	80%	8 mL
Equilibration solution (ES)				
Ethylene glycol	Millipore-Sigma	#109621	7.5%	0.75 mL
DMSO	Sigma	#D2650-100 mL	7.5%	0.75 mL
KOSR	Invitrogen	#10828010	20%	2 mL
DMEM F/12 (15 mM HEPES)	Invitrogen	11330032	65%	6.5 mL
Vitrification solution (VS)				
Ethylene glycol	Millipore-Sigma	#109621	15%	1.5 mL
DMSO	Sigma	#D2650-100 mL	15%	1.5 mL
KOSR	Invitrogen	#10828010	20%	2 mL
Sucrose	Fisher	#AAJ6514836	0.5 M	
DMEM F/12 (15 mM HEPES)	Invitrogen	11330032		Fill to 10 mL
Rewarming solution (RS)				
KOSR	Invitrogen	#10828010	20%	2 mL
Sucrose	Fisher	#AAJ6514836	1 M	
DMEM F/12 (15 mM HEPES)	Invitrogen	11330032	Fill to 10 mL	Fill to 10 mL
Diluent solution (DS)				
KOSR	Invitrogen	#10828010	20%	2 mL
Sucrose	Fisher	#AAJ6514836	0.5 M	
DMEM F/12 (15 mM HEPES)	Invitrogen	11330032	Fill to 10 mL	Fill to 10 mL

* ≤ 0.05, ** ≤ 0.01, *** ≤ 0.001, **** ≤ 0.0001.

#### 2.3.2 Shipping vitrified organoids

For information on how to prepare the vapor shipper dewar (IVF Store, #10817330) and the protective shipping container (IVF Store, #20750409) for organoid shipment, please refer to the manufacturer’s operation manual and add canes containing Cryolocks^®^ right before shipping. The dewar should maintain its temperature for 21 days after removing the LN_2_. Overnight shipping arrangements should be made accordingly.

#### 2.3.3 Immunocytochemistry

The hCOs were fixed at day 30 with 4% paraformaldehyde (PFA; Fisher, #AA433689M) in PBS for 30 min at 4 °C, except that day 90 hCOs were fixed for 1 h in 4% PFA at 4 °C. Organoids were then washed 3 times for 10 min in 1 × PBS and incubated overnight in 30% sucrose in 1 × PBS at 4 °C. Organoids were embedded using O. C. T. (Thermo, #23–730-571) in Tissue-Tek Cryomolds (Sakura, #32663) and frozen on dry ice before being stored at −80 °C. The blocks were sectioned on a cryostat (Leica) at 20 μm thickness. After 2 h of drying at RT, the sections were outlined using a hydrophobic A-PAP Pen (Ted Pella, #22309). Once the ink had dried the slides were washed 3 times, 5 min each, with PBS. The tissue was permeabilized with 0.2% Triton-X100 (Sigma, #T8787-250ML) for 20 min at RT, followed by incubation in blocking solution: 1 × PBS containing 5% normal goat serum (Thermo, #16210–072), 1% BSA (Sigma, #A3912-100G), and 0.05% Tween-20 (Sigma, #P2287-100ML) for 1 h at RT. All samples were incubated in primary antibodies (Table 2) in blocking solution overnight at 4 °C. The following day slides were washed 4 × 10 min, with 1 × PBS containing 0.05% Tween-20 (PBST) and incubated for 90 min with secondary antibody (Table 2) in blocking solution. Slides were washed 3 × 10 min, in PBST and then incubated with bisbenzimide (Thermo-Fisher, #H3569) for 5 min for nuclear counterstain. This was followed by 3 × 5 min washes in 1 × PBS, then coverslips were mounted on slides with Glycergel mounting medium (Agilent Dako, #C0563) and allowed to cure at RT O/N. The following day the coverslip edges were sealed with clear nail polish (EMS, #72180) and stored at 4 °C.

**Table 2:** Antibodies.

Antibody	Species	Dilution	Company	Cat#
OCT 3/4	Rt	1 (250)	R and D Biosystems	#MAB1759
NANOG	Rb	1 (500)	Abcam	#ab21624
CTIP2/ BCL11B	Rt	1 (300)	Abcam	#ab18465
GFAP	Rb	1 (2,000)	Dako	#Z0334
SATB2	M	1 (100)	Abcam	#ab51502
TUBB3/TuJ1	M	1 (1,000)	Covance	#MMS-435p
TBR2/EOMES	Mouse	1 (400)	R&D	#MAB6166
HOPX	Rb	1 (500)	MilliporeSigma	#HPA030180
MKI67	Mouse	1 (1,000)	BD biosciences	#550609
Anti-active Caspase-3	Rb	1 (1,000)	BD	#559565
SOX2	Rb	1 (1,000)	MilliporeSigma	#AB5603
anti-Mouse IgG Alexa Fluor 568	Gt	1 (500)	Invitrogen	#A-11031
Anti-Rabbit IgG AlexaFluor 647	Gt	1 (500)	Invtirogen	#A-21245
Anti-Rat IgG AlexFluor 488	Gt	1 (400)	Invitrogen	#A-11008

#### 2.3.4 Organoid size and fluorescence intensity quantification and microscopy

To measure the size of organoids after vitrification and rewarming, individual organoids were placed into a low attachment U-Bottom 96-well plate and imaged using an Incucyte live imaging system (Sartorius) from days 0–10 post-rewarming (day 21–30 p.d.). Phase contrast images were taken at 10 × magnification using the spheroid protocol. For area measurements after day 30 (removed from 96 well plate), organoids were imaged on an EVOS epifluorescence microscope (Thermo) at 4X magnification. Organoid area was measured in Fiji (ImageJ) by setting the scale based on the scale bar and tracing the perimeter of the organoid with the free-hand tool. ICC images for quantifications were acquired on a DMI6000B epifluorescence microscope (Leica) equipped with a Hamamatsu Photonics Camera (#C4742-80-12AG). Representative images of individual organoids were taken under 20 × magnification and were quantified for fluorescence intensity using Fiji based on a protocol described in Dang et al. (2021). Briefly, 10 × magnification images were taken of each organoid containing as much of the organoid as possible. For nuclear proteins (MKI67, Cleaved Caspase-3[Casp3], CTIP2/BCL11B, SATB2, SOX2, and TBR2/EOMES), the percentage area immunolabeled (% Area) was calculated by measuring the area that stained positive for each of the above markers that overlapped with bisbenzimide/DNA labeling. Bisbenzimide was used to create a region of interest (ROI) and any nuclear stain that fell within the ROI was counted as positive for one of the above markers. For cytoplasmic proteins (TUBB3/TuJ1, HOPX, GFAP) the whole organoid was set as the ROI. The relative intensity for immunolabeling was calculated for each cytoplasmic protein by dividing the mean intensity of immunoreactivity for the protein of interest by the mean fluorescence intensity of bisbenzimide within the same ROI. Representative images of all hCOs were taken using a Fluoview FV3000 confocal microscope (Olympus).

#### 2.3.5 RNA extraction and RT-qPCR

Total RNA was isolated with the RNeasy Mini Kit (Qiagen, #74104) according to the manufacturer’s protocol. cDNA was synthesized with the SuperScript™ III First-Strand Synthesis SuperMix kit (Invitrogen, #18080–051). The RNA integrity values (260/280 and 260/230) were equivalent between vitrified and control organoids. RT-qPCR was carried out with Power SYBR Green PCR Master Mix (Applied Biosystems, #4367659) using primers listed in Table 3. qPCR was performed on a Quantstudio 3 Real-Time PCR System (Thermofisher). Fold change was calculated using the 2^−ΔΔCt^ method: $\begin{array}{l} \Delta \Delta \mathrm{Ct}=(\begin{array}{l} \mathrm{Ct}\;\text{gene of interest}\;[\text{Vitri or ctrl}]\\ -\text{average}\;\mathrm{Ct}\;\text{GAPDH} \end{array})\\ -(\text{average}\Delta \mathrm{CT}\;\text{ctrl gene of interest}) \end{array}$

$$\begin{array}{l} \mathrm{eg}.\text{Vitrified}\Delta \Delta \mathrm{Ct}\;[\mathrm{SOX}2]=(\mathrm{CT}\;\mathrm{SOX}2-\mathrm{CT}\;\text{GAPDH})-\\ \;\;\;\;\;\;\;\;\;\;\;\;\;\;\;\;\;\;\;\;\;\;\;\;\;\;\;\;\;\;\;\;\;\;\;\;\;\;\;\;\;\;\;\;\;\;\;\;\;\;\;\;\;\;\;\;\;\;\;\;\;\;\;\;\;\;(\mathrm{avg}.\Delta \mathrm{CT}\;\text{control}\;\mathrm{SOX}2) \end{array}$$

**Table 3:** Primers.

Name	Forward sequence	Reverse sequence
*GAPDH*	ATGTTCGTCATGGGTGTGAA	GGTGCTAAGCAGTTGGTGGT
*MKI67*	ACGCCTGGTTACTATCAAAAGG	CAGACCCATTTACTTGTGTTGGA
*SOX2*	TACAGCATGATGCAGGACCA	TCATGTAGGTCTGCGAGCTG
*CTIP2/*BCL11B	GAGTACTGCGGCAAGGTGTT	TAGTTGCACAGCTCGCACTT
*SATB2*	CGACGGATGGTACAGAGGTT	GCCAACATCAACATCACAGC
*TBR2/EOMES*	CACCGCCACCAAACTGAGAT	CGAACACATTGTAGTGGGCAG
*HOPX*	CAGCTAGCAATGGAACATACAA	AAGGCCTGCCCTCAGAGT
*TUBB3/TuJ1*	CATTCTGGTGGACCTGGAAC	CCTCCGTGTAGTGACCCTTG
*GFAP*	GTGCAGACCTTCTCCAACCT	CACCACGATGTTCCTCTTGA

#### 2.3.6 Electrophysiological recordings

##### Multielectrode array recordings

At day 90 post derivation (p.d.), SOSR-COs were plated on MEA plates to observe activity using a published protocol (Pan et al., 2024) with minor modifications. Briefly, 6-well MEA plates (Axion Biosystems, #M384-tMEA-6 W) were washed with 1 mL of 1% Tergazyme (Alconox, #1325) in ddH_2_O followed by 3 washes with ddH_2_O. One milliliter of organoid culture medium was added to each well and placed in a 37 °C incubator for 2 days. Media was aspirated and replaced with 0.1% polyethylenimine (PEI; Sigma, #P3143-100 mL) in ddH_2_O and incubated at 37 °C for 1 h. Each well was rinsed 3 × with ddH_2_O and allowed to air dry in the cell culture hood for 1 h at RT with the lid off. Next, we made a 1:10 mixture of Geltrex in DMEM/F12 and added 50 μL to the center of each well. The plates were left in the incubator O/N at 37 °C. The Geltrex solution was then aspirated and any Geltrex covering the electrode contacts was sprayed away using DMEM/F12 and a p1000 pipette. One to two organoids/treatment/cell line were transferred to each well of the MEA plate. The residual media was aspirated with a p200 tip, and the plate was placed in the 37 °C incubator for 5 min. The plate was then removed and a single drop of 100% Geltrex was applied to the top of each organoid using a p200 pipette tip. The plate was then placed back in the incubator for another 10 min. Culture media (250 μL) was added gradually over several hours. The next day the remaining 750 μL of media was added and the plate was left in the 37 °C incubator for 2 days. 3 N media was gradually transitioned over the next week to BrainPhys™ media (STEMCELL Tech., #05790). Recordings were taken the day after media changes, which were performed twice per week using an Axion Maestro Original running Axis Software. Recordings were made using 200 Hz – 3 kHz bandpass filtering and analyzed using the Axis Metrics Plotting Tool (Axion Biosystems).

##### Whole cell patch clamp recordings

Day 90 SOSR-COs were transitioned from 3 N media to BrainPhys™ media over 1 week and cultured until day 200. Organoid slice preparation and recordings were modified from our prior work (Tidball et al., 2023). Briefly, SOSR-CO slices (~300–400 μm) were cut manually in ice-cold, oxygenated “slicing” solution saturated with 95% O_2_/5% CO_2_ because the organoids were too small to cut with an oscillating tissue slicer. The slicing solution contained (in mM): 110 sucrose; 62.5 NaCl; 2.5 KCl; 6 MgCl_2_; 1.25 KH_2_PO_4_; 26 NaHCO_3_; 0.5 CaCl_2_ and 20 D-glucose (pH 7.35–7.4 when saturated with 95% O_2_/5%CO_2_ at RT of 22-25 °C). Slices were incubated in slicing solution for 20–30 min at RT and then in a 1:1 mixture of slicing solution and artificial cerebrospinal fluid [ACSF, containing (in mM): 125 NaCl; 2.5 KCl; 1 MgCl_2_; 1.25 KH_2_PO_4_; 26 NaHCO_3_; 2 CaCl_2_ and 20 D-glucose, (pH 7.35–7.4)] in a holding chamber aerated continuously with 95% O_2_/5%CO_2_ at 35 °C for 30 min before being transferred to ACSF at RT. Individual slices were transferred to a recording chamber perfused (2–3 mL/min) with ACSF bubbled continuously with 95% O_2_/5% CO_2_ at RT. Cells were visualized and selected using an E600FN upright microscope (Nikon) with a Namarski 40 × water immersion objective. For action potential recordings, recording electrodes had a resistance of 4–6 MΩ when filled with a potassium gluconate (K-gluconate)-based pipette solution consisting of (in mM): 140, K-gluconate; 4, NaCl; 0.5, CaCl_2_; 10, HEPES; 5, EGTA, 5 phosphocreatine; 2, Mg-ATP and 0.4, GTP (pH 7.2–7.3 adjusted with KOH). Whole cell current-clamp recordings were used to measure APs. Repetitive firing was evoked by a series of 1,500 ms depolarizing currents varying from −60pA to 300pA in 10pA steps from the resting membrane potential. Spontaneous firing of APs from their resting membrane potential was recorded using a gap-free mode. For recording sEPSCs in the same cells, recordings were switched to whole cell voltage-clamp recording mode. sEPSCs were recorded at a holding potential of −70 mV in the presence of 10 μM bicuculline in the external solution to block GABAergic synaptic responses. The signals were amplified using a Multiclamp700B amplifier (Molecular Devices), filtered at 2–4 kHz and digitized at 20 Hz for analysis. Data were acquired with a Digidata1440 digitizer and analyzed using pClamp11.3.

##### Statistics

Data were organized and analyzed in Excel (Microsoft). All graphs and statistics were performed in Prism 10 (Graphad). Cell counts, qPCR, and sEPSP amplitude and frequency results were tested for statistically significant differences using students *t*-test or one-way ANOVA with Dunnett’s post-test. Size measurements, MEA recordings and input–output curves were analyzed using two-way ANOVA with Šídák post-test. Error bars depict the standard error of the mean (SEM) (see the Supplementary Table 1 for the list of tests used).

## 3 Results

### 3.1 Vitrification is capable of cryogenically preserving human cortical organoids

**Figure 2:**
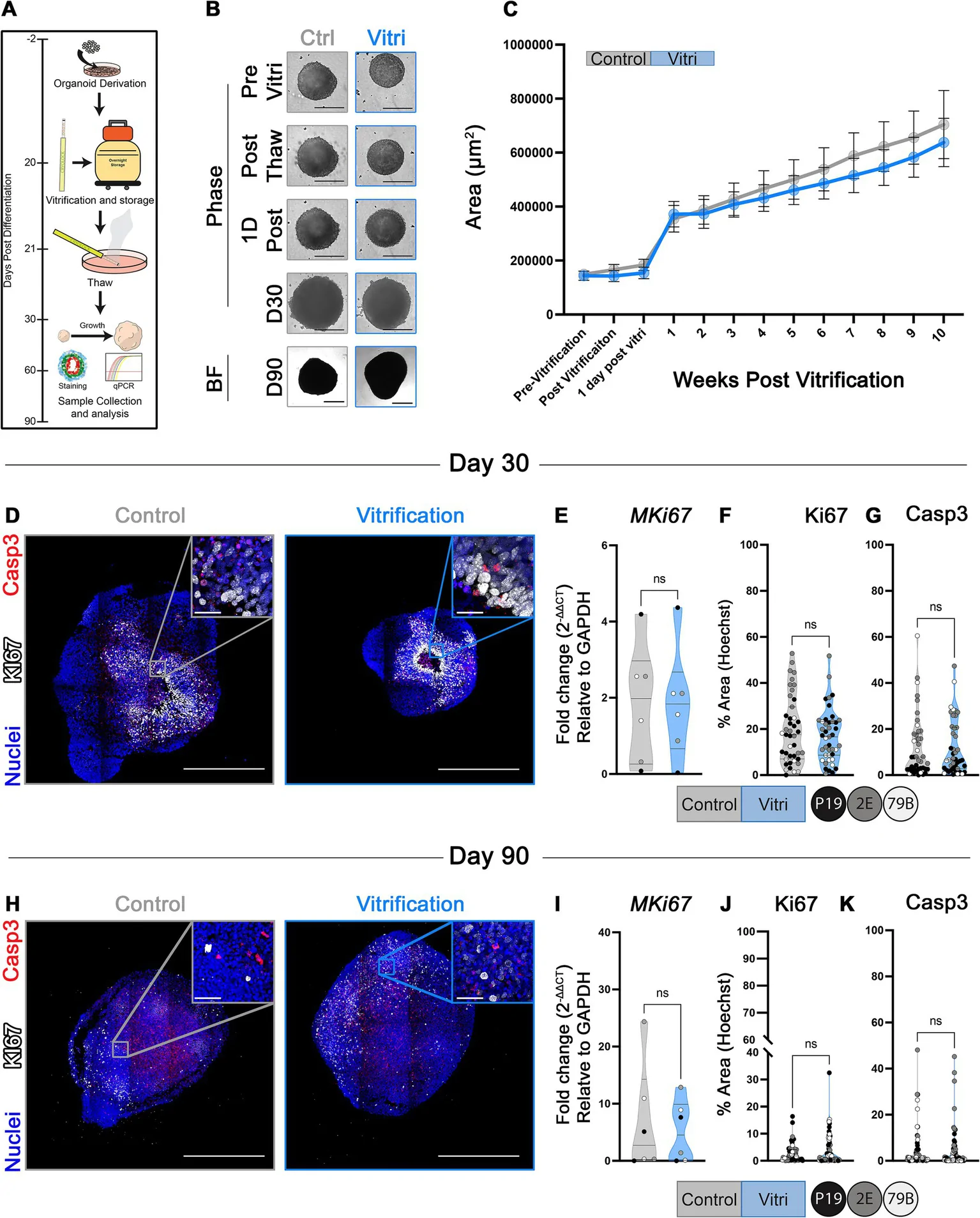
Vitrification does not impact organoid growth, cell proliferation or cell death. **(A)** Schematic depicting the workflow with SOSR-COs cultured to day 20 p.d. and then half are vitrified and rewarmed the next day (Vitri group), while the other half (control group) remained in culture. Control and Vitri SOSR-COs were then collected and analyzed at 30 or 90 days p.d. **(B)** Representative phase and brightfield (BF) images of control and Vitri SOSR-COs before (pre-Vitri) or 0, 1, 9 and 70 days post vitrification (days 20, 21, 22, 30, and 90 p.d.). Scale bar = 400 μm (phase images) or 500 μm (BF images). **(C)** Control (gray) and Vitri (blue) SOSR-CO growth (μm^2^) was monitored for 10 days, beginning on the day of rewarming. No significant difference is seen in SOSR-CO growth between groups [*F*(12, 109) = 0.09, *p* > 0.99]. *N* = 6 experiments for weeks 1–10 p.v. *N* = 3 for Pre-, day 0 post-, 1 day post-vitrification 10–24 hCOs/line/experiment. **(D)** Representative images of day 30 p.d. control and Vitri SOSR-COs immunolabeled for MKI67 (gray), Casp3 (red), and Hoechst (blue). **(E–G)** No differences are observed between groups for the cell proliferation marker MKI67 quantified using RT-qPCR **(E)** (*p* = 0.99) and IF [**(F)**; *p* = 0.38], or for Casp3 IF (*p* = 0.91). Black, gray and white dots represent data points from the 3 different hPSC lines (P19, 2E and 79B, respectively). **(H–K)** Proliferation (MKI67) and cell death (Casp3) were evaluated in Control (gray) and Vitri (blue) SOSR-COs at 90 days p.d. **(H)** Representative confocal images of MKI67 (gray) and Casp3 (red) immunolabeled hCOs. MKI67 is unchanged between groups using RT-qPCR [**(I)**; *p* = 0.71] and IF [**(J)**; *p* = 0.27], and Casp3 IF is also unchanged [**(K)**; *p* = 0.86]. Statistical tests: 2-way ANOVA **(C)**; Unpaired *t*-test **(E–G,I–K)**. Error bars in **(D)** represent SEM. Scale bars for IF images: 500 μm (large), 50 μm (inset). IF: *N* = 3 experiments with 2–8 hCOs/line. Each point represents a single hCO. For qRT-PCR, each point represents a single cell line in one experiment using 3–5 hCOs/line/experiment.

To test whether vitrification would successfully preserve brain organoids, we chose to vitrify hCOs at day 20 post-differentiation (p.d.) to allow them to fully self-organize while still being small enough to facilitate easy penetration of the CPAs. We patterned human iPSC lines into “dorsalized” hCOs using the self-organizing single-rosette cortical organoid (SOSR-CO) protocol (Tidball et al., 2023). On day 20, the organoids were separated into two groups: one underwent cryopreservation using our vitrification protocol (referred to as Vitri), which entails treating the organoids with increasing concentrations of the cryopreservatives sucrose, DMSO, and ethylene glycol followed by immediate exposure to −195 °C (Figures 1, 2A; Supplementary experimental procedures 1–11). The other group remained in culture (referred to as Control). One day post-vitrification (p.v.), hCOs were warmed through immediate exposure to 37 °C rewarming solution (RS) followed by washes in decreasing levels of CPAs (Figures 1, 2A; Supplementary experimental procedures 12–21) and placed back into their usual medium alongside age-matched controls. SOSR-COs were analyzed for growth and cell composition at two timepoints (Figure 2A). To ensure organoid cryopreservation was not influenced by neighboring organoids, we evenly spaced our day 20 organoids (143,772 μm^2^ ± 16,940 μm^2^ SEM) (Figure 2C; Supplementary statistics table) along the length of the Cryolock^®^, leaving room for 4–6 organoids.

For the first 10 days post vitrification (p.v.) (day 21–30 p.d.), the size of the SOSR-COs was monitored. We found that rewarming hCOs after vitrification did not cause any growth deficit (Figures 2B,C). To assess differences in proliferation and apoptosis, we used immunofluorescence (IF) quantifications of MKI67 and cleaved caspase 3 (Casp3), respectively, with Fiji based on a previously described protocol (Dang et al., 2021). We also used qRT-PCR to measure MKI67 transcript levels. At 2 and 10 days p.v. (days 22 and 30p.d.), we found no significant differences in cell proliferation (MKI67^+^) or apoptosis (Casp3^+^) between vitrified and control SOSR-COs (Figures 2D–G and Supplementary Figures 2A–C). At day 90 p.d. (70 days p.v.), Vitri SOSR-COs maintained the same levels of cell proliferation (MKI67^+^) and apoptosis (Casp3^+^) as controls (Figures 2H–K).

**Figure 3:**
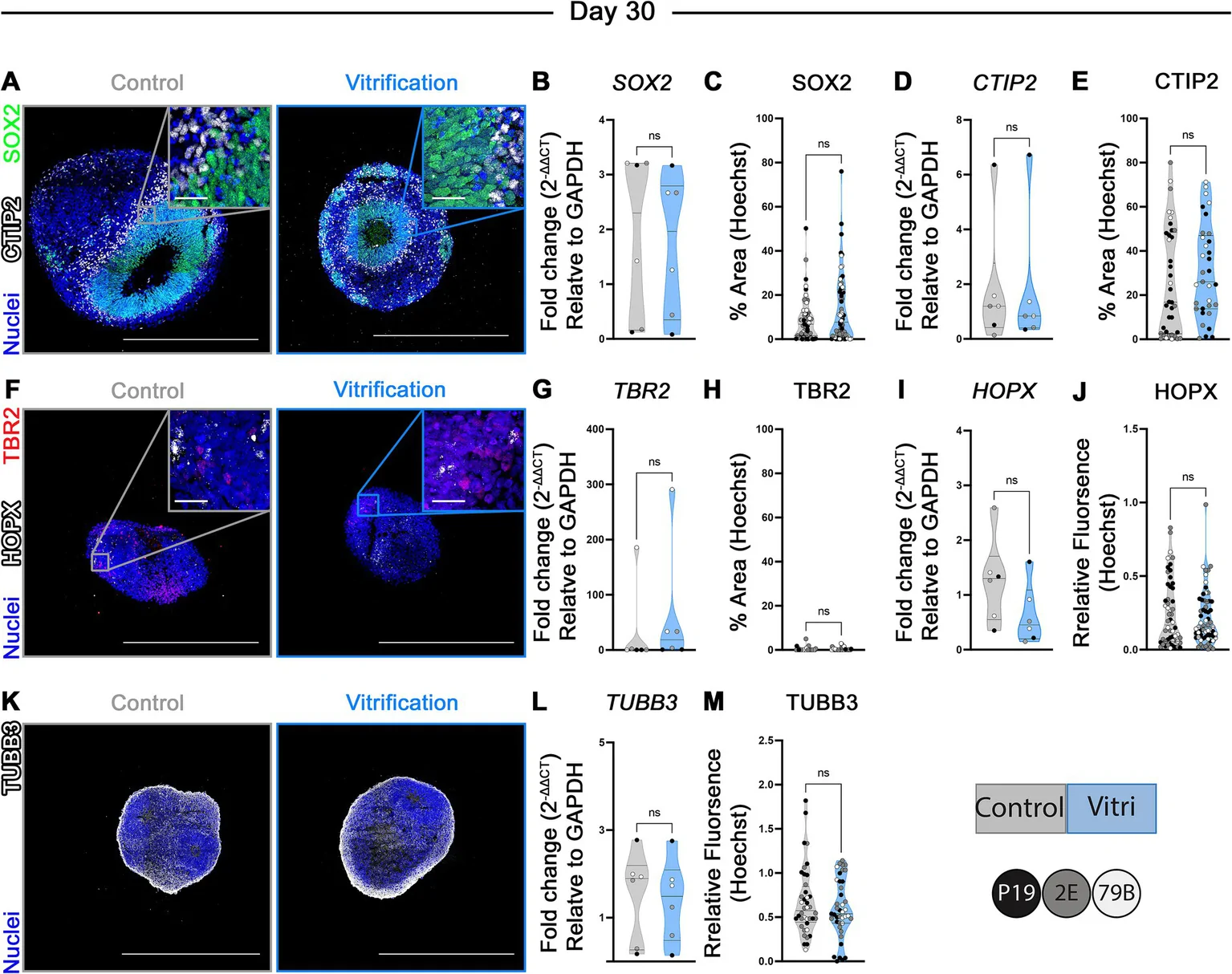
SOSR-COs at 10 days post-vitrification have similar cellular composition to control organoids. **(A)** Representative confocal images of control (left) and vitri (right) organoids immunolabeled for the neural stem/progenitor cell marker SOX2 (green) and deep layer neuron marker CTIP2 (gray). **(B,C)** SOX2 expression measured by RT-qPCR [**(B)** *p* = 0.84] or IF [**(C)** *p* = 0.4] do not differ between vitri (blue) and control (gray) groups. **(D,E)** Similarly, no changes are found in CTIP2 (gray) expression [**(D)** *p* = 0.96] or IF [**(E)** *p* = 0.4]. **(F)** Representative confocal images of control and vitri SOSR-COs immunolabeled for IPC and oRG cell markers TBR2 (red) and HOPX (gray), respectively. **(G–J)** Both TBR2 and HOPX expression are equivalent between groups by RT-qPCR [TBR2 in **(G)** *p* = 0.61; HOPX in **(I)** *p* = 0.14] and IF [TBR2 in **(H)** *p* = 0.9; HOPX in **(J)** *p* = 0.19]. **(K)** Representative confocal images of hCOs immunolabeled for the neuronal marker TUBB3 (gray). **(L,M)** Beta-III-tubulin expression (TUBB3) by RT-qPCR [**(L)** *p* = 0.84] and IF [**(M)** *p* = 0.33] is unchanged between controls (gray) and vitri (blue) groups at day 30. Hoechst staining of nuclei in blue for panels **(A,F,K)**. The 3 different human pluripotent stem cell (hPSC) lines are shown in the plots as different colored dots (2E, gray; 79B, white; P19, black). Statistics: Unpaired *t*-test. Scale bars: 500 μm (large), 50 μm (inset). IF: *N* = 3 experiments with 2–8 hCOs/line. Each point represents a single hCO. For qRT-PCR, each dot represents a cell line in one experiment with 3–5 hCOs/line/experiment.

We also assessed whether vitrification impacted the cellular composition of vitrified SOSR-COs. At day 30 p.d. we did not observe any significant difference in the number of neural stem/progenitor cells (SOX2^+^) or deep layer (CTIP2/BCL11B^+^) neurons between vitrified and control SOSR-COs (Figures 3A–E). We also found an equivalent number of intermediate progenitor cells (IPCs) marked by TBR2/EOMES^+^ and HOPX^+^ outer radial glia (oRG) (Figures 3F–J). Neurons (TUBB3/TuJ1^+^) were also found to be equivalent between vitrified and control SOSR-COs (Figures 3K–M). These data indicate that vitrification of SOSR-COs at day 20 p.d. does not impact progenitor or neuronal cell numbers after culture for 10 days post-rewarming.

**Figure 4:**
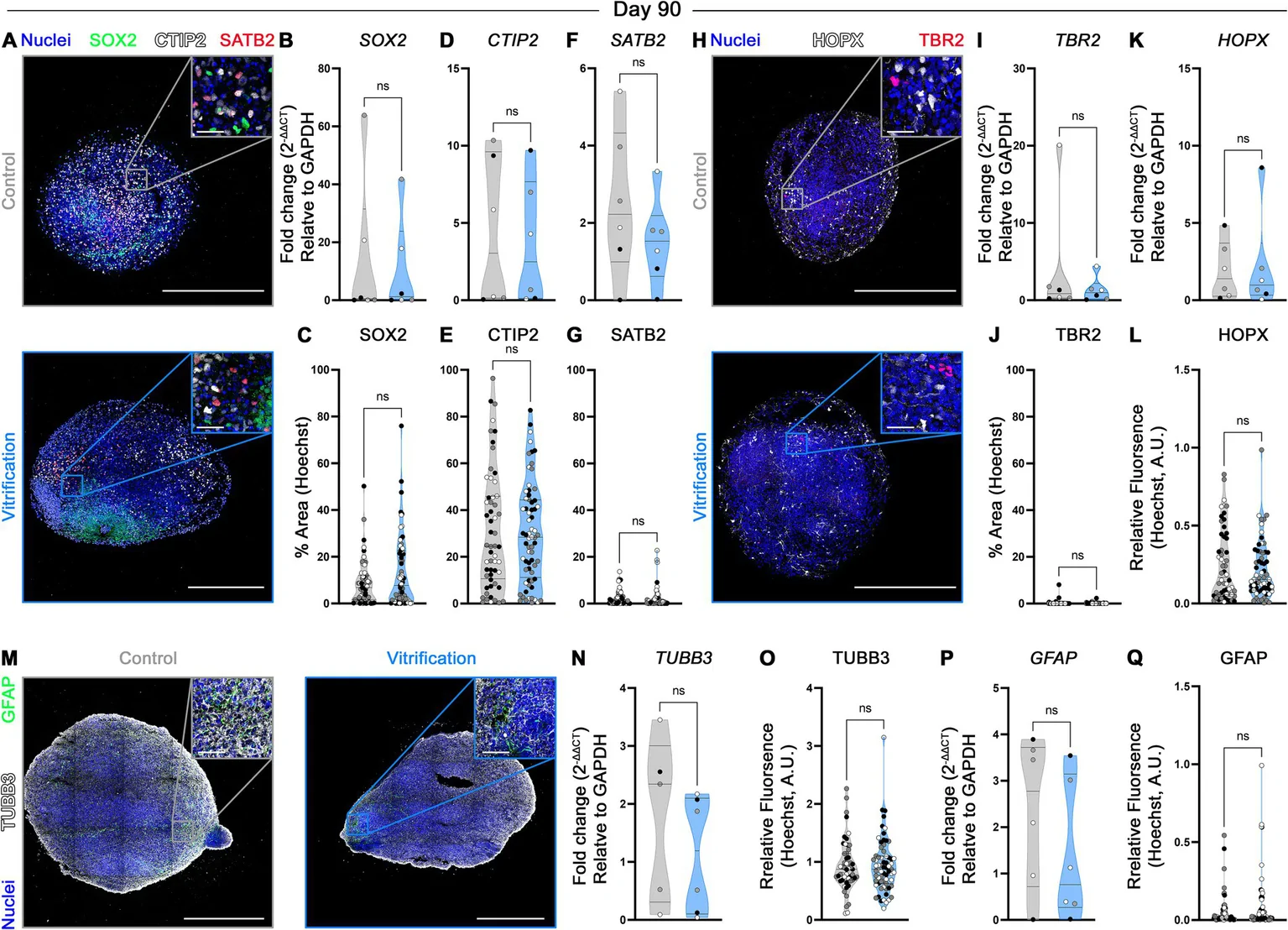
Vitrified SOSR-COs develop similarly to unvitrified controls. **(A)** Representative confocal images of day 90 p.d. control (top) and Vitri (bottom) SOSR-COs immunolabeled for the neural stem/progenitor cell marker SOX2 (green), deep layer neuron marker CTIP2 (gray), and superficial layer neuron marker SATB2 (red). Hoechst nuclear stain is in blue. **(B,C)** SOX2 is unchanged between control (gray) and Vitri (blue) groups by RT-qPCR or IF [**(B)**; *p* = 0.76; **(C)**; *p* = 0.08]. **(D,E)** CTIP2 expression by RT-qPCR and IF [**(D)**; *p* = 0.8; **(E)**; *p* = 0.73] are unaffected by vitrification. **(F,G)** Levels of SATB2 by RT-qPCR and IF [**(F)**; *p* = 0.29; **(G)**; *p* = 0.72] do not significantly differ between groups. **(H)** Representative confocal images of control and Vitri SOSR-COs immunolabeled for TBR2 (red) and HOPX (gray), with Hoechst nuclear stain (blue). **(I–L)** The expression of both TBR2 [**(I)**, RT-qPCR, *p* = 0.44; **(J)**, IF, *p* = 0.39] and HOPX [**(K)**, RT-qPCR, *p* = 0.86; **(L)**, IF, *p* = 0.34] is unchanged by vitrification. **(M)** Representative confocal images of the neuronal marker TUBB3 (gray) and astrocyte marker GFAP (green). **(N,O)** TUBB3 expression by qRT-PCR and IF intensity is unchanged between controls (gray) and Vitri (blue) SOSR-COs at day 90 (RT-qPCR, *p* = 0.39; IF, *p* = 0.34). **(P,Q)** No significant differences are found between the mRNA expression or IF intensity of GFAP^+^ cells between vitrified and control samples [**(Q)**, RT-qPCR: *p* = 0.32; **(R)**, IF: *p* = 0.35]. Statistics: Unpaired *t*-test **(B–G,I–L,N–Q)**. Scale bar: 500 μm (large), 50 μm (inset). IF: *N* = 4 experiments with 3–8 hCOs /line. Each point represents a single hCO. qRT-PCR used 3–5 hCOs/line. Each point represents a line in a single experiment.

To evaluate the potential effect of vitrification on maturity at a later stage of hCO development, we examined day 90 p.d. (70 days p.v.) SOSR-COs for cell type diversity in a similar manner. Day 90 p.d. vitrified SOSR-COs showed no difference in transcript levels or immunolabeling for the neural stem/progenitor cell markers SOX2^+^, TBR2^+^ or HOPX^+^ (Figures 4A–C,H); for CTIP2^+^, SATB2^+^, and TUBB3^+^ neuronal populations (Figures 4A,D–G,M–O), or in the number GFAP^+^ labeled astrocytes (Figures 4M, P, Q). Together, these data indicate that vitrification does not impact early and late stages of SOSR-CO development.

**Figure 5:**
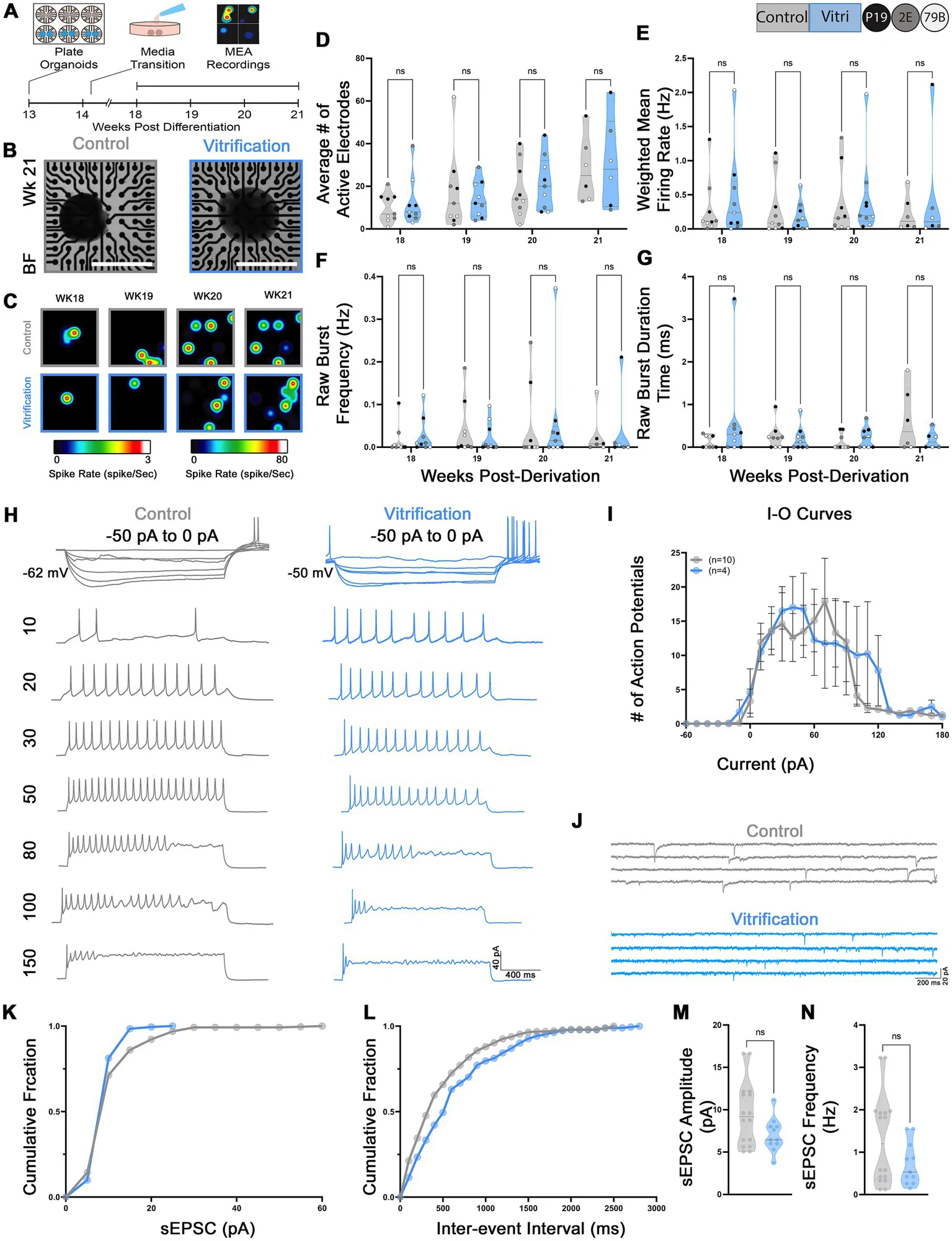
Vitrified hCOs have similar electrical properties. **(A)** Schematic showing the protocol for hCO recordings on 6-well MEA plates. **(B)** Brightfield images of representative SOSR-COs in 1 well of a 6-well MEA plate (scale bar 1,250 μm). **(C)** Representative MEA activity heatmap measured as spikes/s. Week 18–19 scale 0–3 spikes/s, week 20–21 scale 0–80 spikes/s. **(D–G)** Weeks 18–21 SOSR-COs show equivalent numbers of active electrodes [**(D)**, *F*(3, 58) = 0.2, *p* = 0.86], spontaneous field potentials displayed as weighted mean firing rate in Hz **(E)**, [*F*(3, 58) = 0.3, *p* = 0.76], burst frequency **(F)**, [*F*(3, 58) = 0.2, *p* = 0.87], and burst duration **(G)**, [*F*(3, 58) = 1.9, *p* = 0.12] between control (gray) and Vitri (blue) hCOs. *N* = 3 batches with 1–2 organoids/line/batch. Each point represents one line in one experiment. **(H)** Representative traces of evoked APs recorded by whole-cell patch clamp. **(I)** Input–output **(I–O)** curves show similar levels of activity in neurons from control (gray, *n* = 10 cells) and Vitri groups [blue, *n* = 4 cells; *F*(31, 379), *p* = 0.99], *N* = 3 batches with 3–4 organoids. **(J)** Representative traces depicting similar levels of sEPSCs in voltage-clamp recordings of hCO slices made from the two groups. **(K,L)** Graphs depict the cumulative fraction of spontaneous excitatory postsynaptic current (sEPSC) amplitude (pA) and inter-event interval (ms). **(M,N)** Both sEPSC amplitude (pA; *p* = 0.58) and frequency (Hz; *p* = 0.11) are equivalent between control (gray, *n* = 16) and vitrified (blue; *n* = 11) neurons within day 200 SOSR-COs. Statistics: 2-way ANOVA with Šídák post-test **(D–G,I)**; *t*-test **(M,N)**.

In addition to developing a structural and cellular composition that largely recapitulates the developing human cortex, hCOs are also capable of exhibiting neuronal and network activity (Lancaster et al., 2013; Watanabe et al., 2017; Sun et al., 2019; Trujillo et al., 2019; Samarasinghe et al., 2021). We assessed activity by plating day 90 p.d. SOSR-COs onto MEAs, slowly transitioning them to BrainPhys medium and then recording spontaneous field potentials (Figure 5A). Between weeks 18–21 we observed an equivalent number of active electrodes and similar levels of firing over a 5-min recording between Vitri and control SOSR-COs (Figures 5B–G). We also maintained separate batches of SOSR-COs in BrainPhys medium until day 200 p.d. and performed whole-cell patch clamp recordings from organoid slices. We found no substantial differences in evoked action potentials (AP) from neurons in control and Vitri SOSR-COs (Figures 5H,I). Although the N is low, our spontaneous excitatory postsynaptic currents (sEPSC) recordings also show comparable amplitude and frequency between control and Vitri neurons (Figures 5J–N), indicating that vitrification does not impede the formation of functional synapses in hCOs.

### 3.2 Vitrification of hCOs derived using a multi-rosette hCO protocol

**Figure 6:**
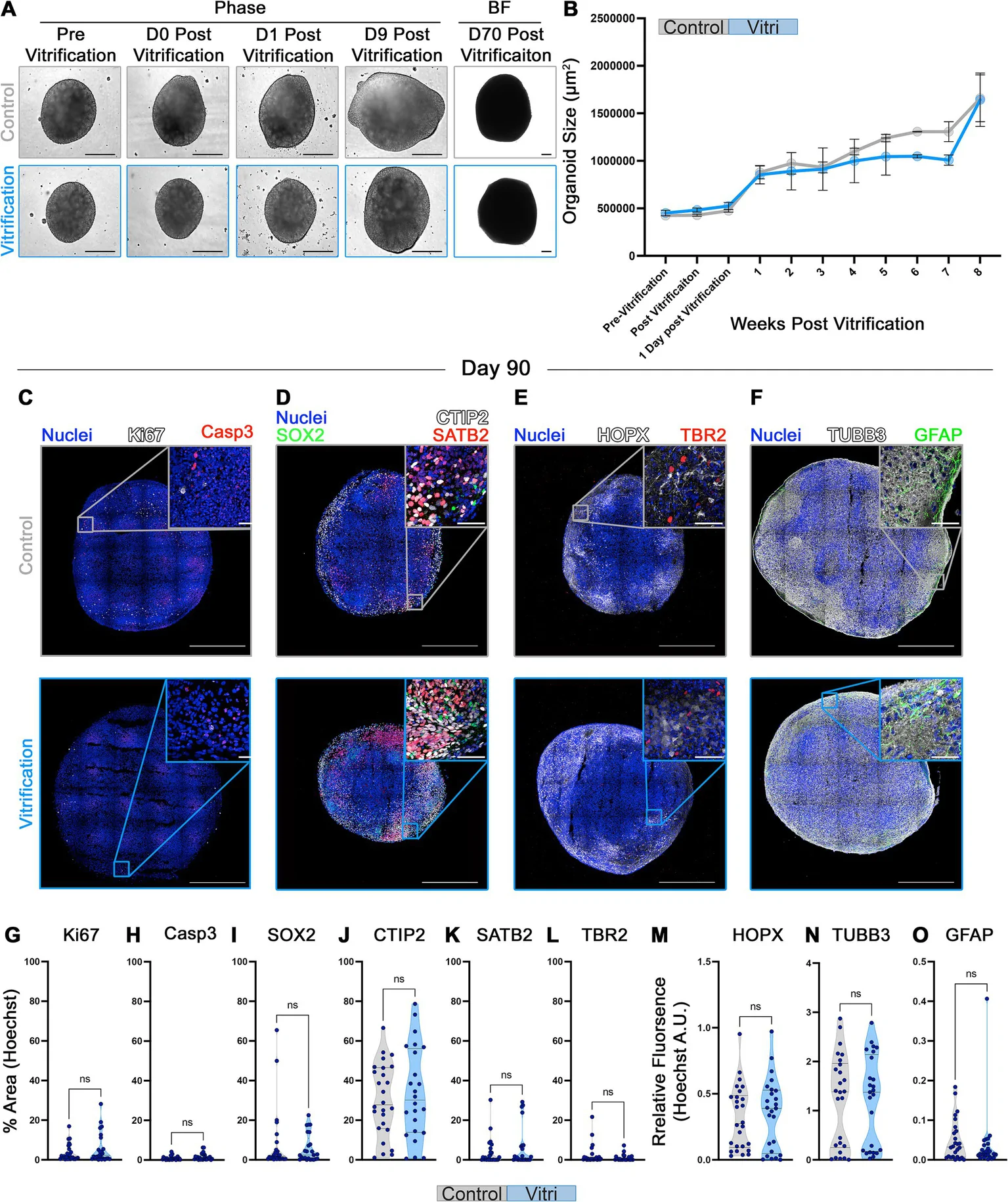
Vitrification can be applied to multi-rosette cortical organoid protocols. **(A)** Representative phase and brightfield (BF) images of control (gray) and Vitri (blue) hCOs generated using the multi-rosette (MR-hCOs) protocol immediately pre and post vitrification (20 and 21 p.d), 1 day post vitrification (22 p.d.), and day 30 and 90 p.d. (10, 70 days p.v.). Scale bar = 400 μm. **(B)** Vitri (blue) MR-hCOs maintained a similar growth rate as controls [gray; *F*(10, 30) = 0.21, *p* = 0.99]. *N* = 2 experiments for pre/0 day/1 day/2–7 week p.v), *N* = 3 experiments (1 and 8 weeks p.v.), with 6–12 hCOs/experiment. **(C–F)** Representative confocal images of 90-day p.d. (day 70 p.v.) control and Vitri MR-hCOs that were immunolabeled with antibodies to **(C)** MKI67 (gray) and Casp3 (red); **(D)** SOX2 (green), CTIP2 (gray) and SATB2 (red); **(E)** TBR2 (red) and HOPX (gray); **(F)** TUBB3 (gray); and GFAP (green). Hoechst nuclear counterstains are in blue. **(G–O)** Vitri (blue) MR-hCOs have the same number of MKI67^+^ **(G)** (*p* = 0.23), Casp3^+^ **(H)** (*p* = 0.88), SOX2^+^ **(I)** (*p* = 0.52), CTIP2^+^ **(J)** (*p* = 0.58), SATB2^+^ **(K)** (*p* = 0.38), TBR2^+^ **(L)** (*p* = 0.20), HOPX^+^ **(M)** (*p* = 0.69), TUBB3^+^ **(N)** (*p* = 0.98) and GFAP^+^ cells **(O)** (*p* = 0.54) compared to unvitrified controls (gray). Statistics: Two-way ANOVA **(B)**, unpaired *t*-test **(G–O)**. Scale bars for IF images: 500 μm (large), 50 μm (inset). IF: *N* = 4 experiments. 4–8 hCOs/experiment. Each point represents a single organoid.

Our SOSR-CO protocol tends to make smaller cortical organoids due to starting with a single rosette. To test whether our vitrification protocol works on a commonly used multi-rosette hCO (MR-hCOs) derivation method that generates larger organoids, we applied vitrification to an hCO protocol developed by Sergiu Paşca’s group (Paşca et al., 2015). MR-hCOs were vitrified on day 20 p.d. (143,772 μm^2^ ± 16,940 μm^2^) (Figure 6B; Supplementary statistics table) as per our previous experiment (Figures 1, 2A). MR-hCOs were rewarmed the day after vitrification and maintained in culture to day 90 p.d. (day 70 p.v.). Similar to our previous results, the MR-hCOs at day 90 p.d. did not show any differences in size (Figures 6A,B), MKI67^+^ proliferating cells, or Casp3^+^ apoptotic cells (Figures 6C,G,H), nor did we observe any difference in the abundance of SOX2^+^ neural stem/progenitor cells, TBR2^+^ IPCs, HOPX^+^ oRG, CTIP2 deep layer, SATB2 superficial layer, TUBB3^+^ neurons or GFAP^+^ astrocytes (Figures 6D–F,I). These data demonstrate our vitrification protocol can be used on multiple organoid protocols and that CPA penetration is not a problem for larger hCOs.

### 3.3 Vitrification of hCOs allows for long-term storage and shipment to other institutions

**Figure 7:**
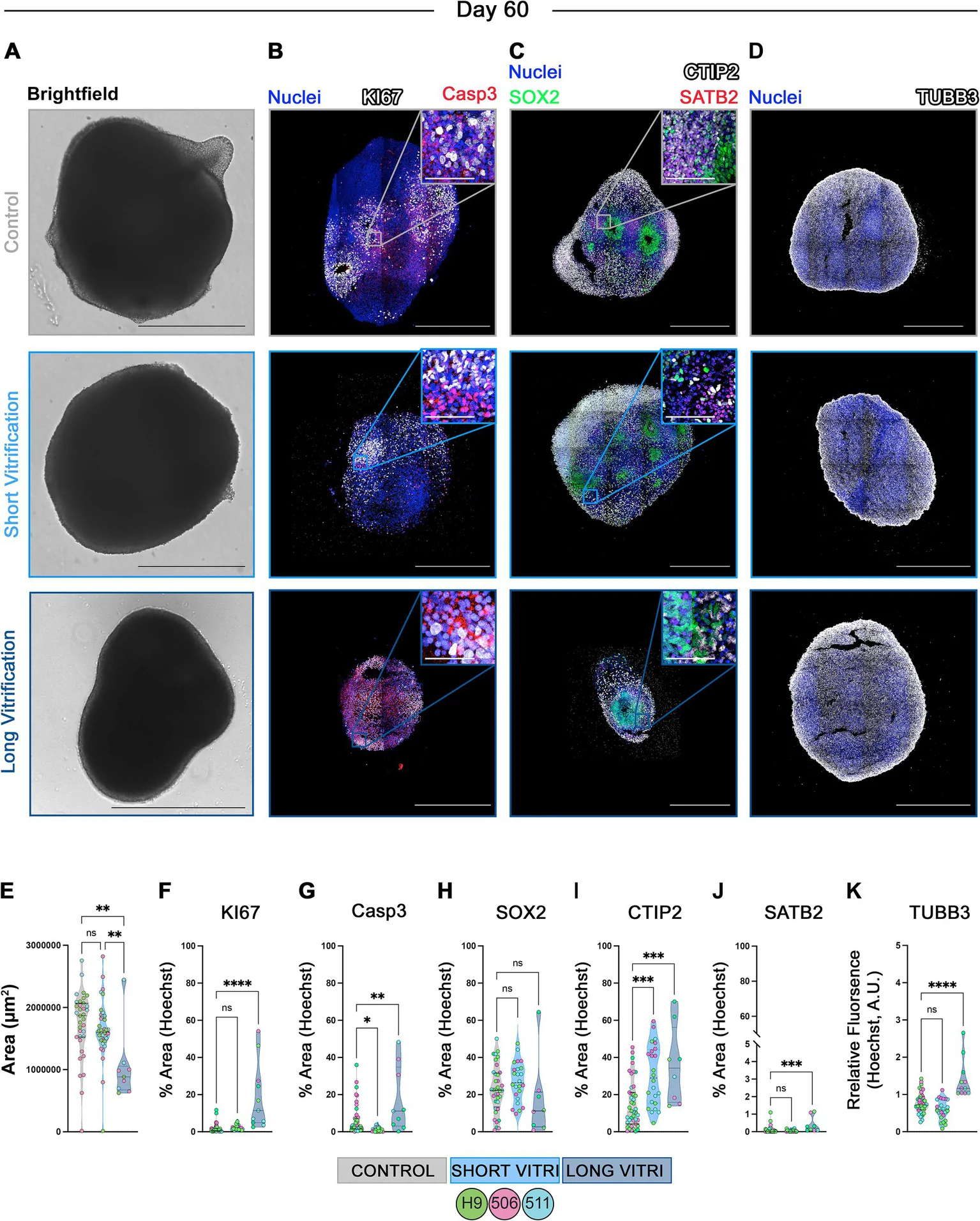
Vitrified SOSR-COs can be stored long-term. The Vitri groups were vitrified on day 35 p.d., stored for over 1 year in LN_2_ (Long-Vitri, dark blue) or overnight (Short-Vitri, light blue), rewarmed and cultured until day 60 p.d. *N* = 2 batches for control and short-Vitri condition; *N* = 1 batch for long-Vitri condition with 1–8 hCOs/line. Each point represents a single hCO. Representative images of organoid size [brightfield in **(A)** and immunofluorescence staining in **(B–D)**]. Controls are significantly larger than the Long-Vitri condition but not the Short-Vitri condition [**A,E**; *F*(2, 78), *p* = 0.76, *p* = 0.0008, respectively]. The Long-Vitri SOSR-COs show significant increases in MKI67^+^ [**(B, F)**, gray; *F*(2, 75) = 28.8, *p* < 0.0001] and Casp3^+^ [**(B,G)**, red; *F*(2, 74) = 12.1, *p* = 0.0016] immunolabeling. SOX2^+^ immunoreactivity was unchanged with either long or short vitrification [**(C,H)**; green; *F*(2, 65) = 1.9, *p* = 0.43], but cells expressing the different neuronal markers, including CTIP2 [**(C,I)**; gray; *F*(2, 72) = 12.4, *p* = 0.0009], SATB2^+^ [**(C,J)**; red; *F*(2, 72) = 9.30, *p* = 0.06], and TUBB3^+^ cells [**(D,K)**; gray; *F*(2, 68) = 23.96, *p* < 0.0001] are significantly increased in the Long-Vitri group compared to controls (gray). SOSR-COs in the short-Vitri condition (light blue) show a significant decrease in Casp3^+^ cells and increase in CTIP2^+^ cells [**(B,G)**, *p* = 0.03; **(C,I)**, *p* = 0.0002, respectively]. However, the numbers of MKI67^+^ **(B,F)**, SOX2^+^ **(C,H)**, SATB2^+^ **(C,J)**, and TUBB3^+^ **(D,K)** cells remain equivalent compared to controls (*p* = 0.98, *p* = 0.69, *p* = 0.4, *p* = 0.08, respectively). *F* statistic for short-Vitri are the same as long-Vitri for corresponding markers. Statistics: One-way ANOVA with Dunnett’s post-test. Scale bar = 500 μm (large), 50 μm (inset) for IF images and 800 μm for brightfield images. * ≤ 0.05, ** ≤ 0.01, *** ≤ 0.001, **** ≤ 0.0001.

As we developed the protocol, we began vitrifying hCOs at various time points for future analysis. We kept in frozen storage a batch that had been vitrified at day 35 p.d. over a year prior that we rewarmed and cultured (Long-Vitri). We retroactively generated two batches of hCOs using the same cell lines and performed vitrification and rewarming a day later as previously described (short-Vitri) alongside unvitrified controls (Control) for comparisons to determine whether vitrified hCOs can be stored over a longer period. After rewarming, SOSR-COs were collected at day 60 p.d. to assess cell type diversity and health. The long-Vitri SOSR-COs demonstrated a significant reduction in size compared to controls (Figures 7A,E). Long-Vitri organoids also show an increase in MKI67^+^ and Casp3^+^ cells, indicating a possible regenerative response. In contrast, no significant difference in the number of MKI67^+^ cells but a reduction in cell death marked by Casp3^+^ appeared in the short-Vitri condition compared to controls (Figures 7B,F,G). We did not observe a significant difference in SOX2^+^ progenitors in either vitrification condition (Figures 7C,H), while the long-Vitri condition displayed a significant increase in TUBB3^+^, CTIP2^+^, and SATB2^+^ neurons. The short-Vitri condition had equivalent levels of TUBB3^+^ and SATB2^+^ neurons but showed an increase in CTIP2^+^ neurons compared to the control (Figures 7C,D,I–K). These results suggest that SOSR-COs stored for at least 1 year survived but displayed some cell type-specific differences at day 60 p.d. that indicate a possible regenerative response. These differences may reflect the effects of longer-term storage, the timing of vitrification, the timepoints selected for vitrification and analysis, or batch to batch variation. Further experiments with larger numbers of hCOs will be needed to provide definitive conclusions.

**Figure 8:**
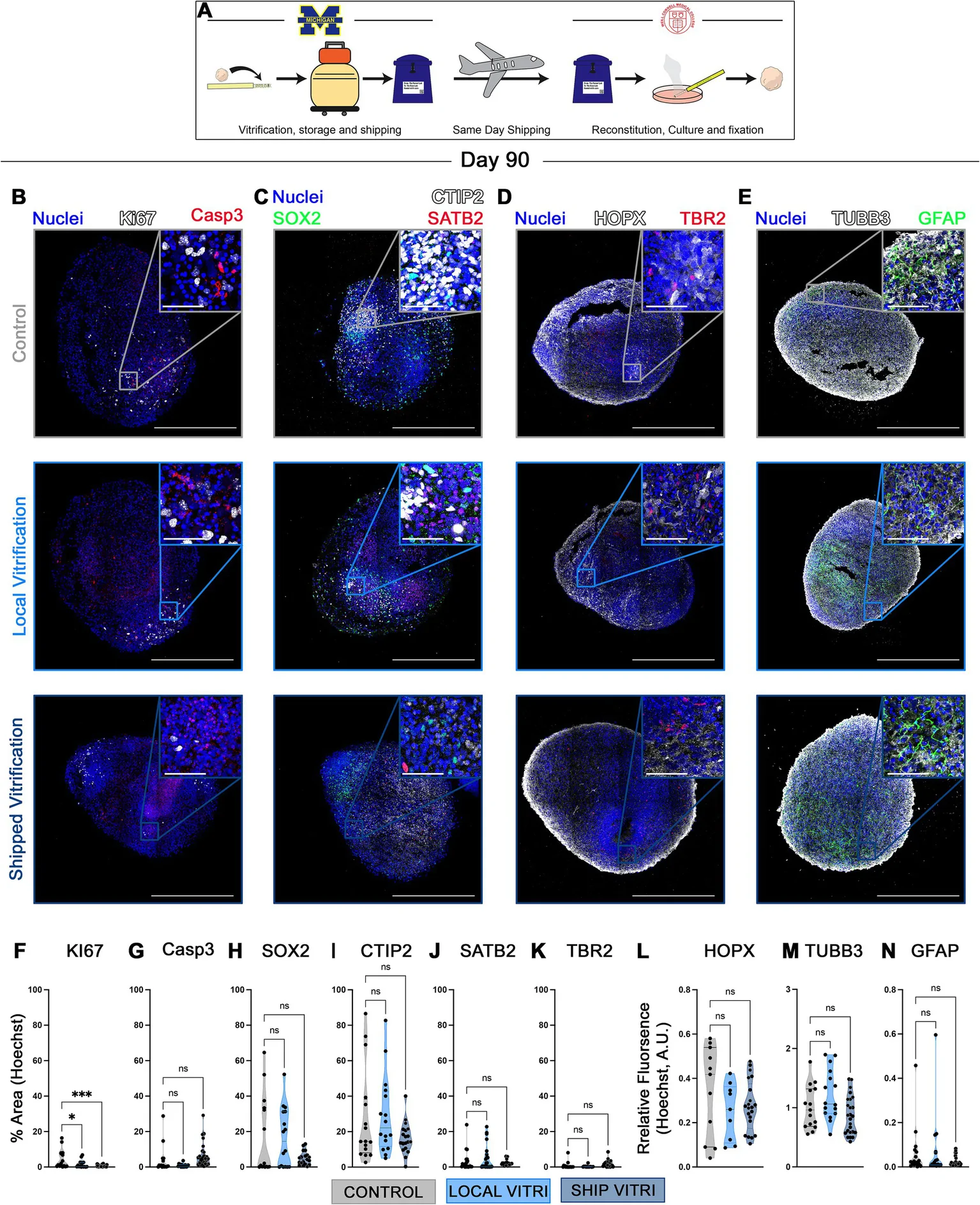
Vitrified organoids can be shipped across the country and reconstituted. **(A)** Graphical representation of experimental design for generating, shipping and reconstituting vitrified SOSR-COs from the University of Michigan to Weill-Cornell Medical Center. Shipped SOSR-COs show a significant reduction in proliferating MKI67^+^ (gray) cells **(B,F)** in both vitrified local (V) and shipped (ShV) hCOs [*F*(2, 58) = 9.12, V *p* = 0.01; ShV *p* = 0.0002]. No differences were found in cells expressing Casp3^+^ (red in **B**). **(G)** [*F*(2, 58) = 4.37, V *p* = 0.29; ShV *p* = 0.29], SOX2^+^ (green in **C**). **(H)** [*F*(2, 47) = 3.27, V *p* = 0.99; ShV *p* = 0.07], CTIP2^+^ (gray in **C**). **(I)** [*F*(2, 48) = 2.13, V *p* = 0.96; ShV *p* = 0.19], SATB2^+^ (red in **C**). **(J)** [*F*(2, _59)_ = 1.52, V *p* = 0.4; ShV *p* = 0.82], TBR2^+^ (red in **D**). **(K)** [*F*(2, 50) = 4.31, V *p* = 0.68; ShV *p* =0.08], HOPX^+^ (gray in **D**). **(L)** [*F*(2, 38) = 1.19, V *p* = 0.27; ShV *p* = 0.35], TUBB3^+^ (gray in **E**). **(M)** [*F*(2, 53) = 4.5, V *p* = 0.21; ShV *p* = 0.38], and GFAP^+^ (green in **E**). **(N)** [*F*(2, 55) = 1.4, V *p* = 0.92; ShV *p* = 0.38]. *N* = 2 batches each with 4–10 hCOs/line. Each point represents a single hCO. Statistics = one-way ANOVA with Dunnett’s post-test. Scale bar = 500 μm (large), 50 μm (inset). *0.05, ***0.001.

A recently published protocol demonstrated that hCOs could be shipped long distances with minimal detrimental effects (Smith et al., 2025). Similarly, we wanted to test whether our cryopreserved SOSR-COs could maintain their viability and structure after long-distance shipping. We vitrified SOSR-COs on day 20 p.d., stored them for 6 months, and then shipped them from the University of Michigan to our collaborators at Weill Cornell Medical School in New York City using overnight delivery (Figure 8A). Our collaborators, new to the vitrification protocol, were then instructed on how to rewarm the organoids using our method. They then cultured these SOSR-COs to day 90 p.d. (70 days p.v.). SOSR-COs were then fixed, embedded and mailed back to the University of Michigan for analysis. The shipped vitrification condition was compared to previously quantified local vitrified (reconstituted 1 day after vitrification) and unvitrified controls derived from the same cell line. In this case both the local and shipped vitrified conditions showed a significant reduction in the number of proliferating cells marked by MKI67 (Figures 8B,F) compared to unvitrified controls. Casp3^+^ cell numbers remained equivalent to controls in both vitrification conditions (Figures 8B,G). SOX2^+^, TBR2^+^, and HOPX^+^ progenitors all remained equivalent to unvitrified controls (Figures 8C,D,H,K,L). Both local and shipped-Vitri conditions showed similar numbers of CTIP2^+^, SATB2^+^, and TUBB3^+^ neuronal populations compared to controls (Figures 8E,I,J,M). Similarly, GFAP^+^ astrocytes also remained equivalent in both vitrification conditions when compared to unvitrified controls (Figures 8E,N). The reduction in MKI67^+^ cells in the vitrification conditions may indicate a slight push toward differentiation but this did not result in a significant increase in mature neurons, leading us to conclude that SOSR-COs can be shipped across the country and reconstituted with minimal impact on hCO development.

## 4 Discussion

The use of vitrification for cryopreserving many different cells and tissues has increased in recent years (Jeong et al., 2020). Previous studies have shown that 2D and 3D cell culture models are amenable to vitrification protocols (Pendergraft et al., 2017; Jeong et al., 2020; Liu et al., 2021; Prinelli et al., 2021; Han et al., 2023; Zolfaghar et al., 2024). Even whole tissues with complex architecture including rat liver and kidney were vitrified and, upon rewarming, showed preserved organ function after transplantation into a host (Han et al., 2023; Sharma et al., 2023). Although brain organoids have been studied for well over a decade (Eiraku et al., 2008; Lancaster et al., 2013), the ability to reliably preserve them has been challenging. In this study we demonstrate that hPSC-derived hCOs, generated using two separate protocols, can be reliably preserved in LN_2_ and rewarmed with limited impact on organoid development and function.

We find that hCOs undergoing vitrification within a wide size range (110617.2 μm^2^–479838.8 μm^2^) (Figures 2C, 6B) survive the rewarming process and show equivalent levels of cell division, cell death and cell-type specific markers compared to unvitrified controls. Additionally, we show that neurons from hCOs that have been vitrified, rewarmed and cultured to relative maturity demonstrate equivalent levels of neuronal activity compared to non-vitrified controls, indicating that the functionality of vitrified hCOs is retained. Our findings also show that this protocol may be applied successfully to a commonly used multi-rosette hCO derivation protocol, suggesting that it will be useful for many neural-based organoid systems. Finally, we have preliminary evidence supporting our protocols ability to store hCOs long-term and that vitrified hCOs can be shipped between institutions with little detriment to the hCO, although we did identify some developmental changes induced by long-term storage and shipping conditions.

Why long-term storage in LN_2_ altered the subsequent development of the hCOs is uncertain at present and may simply reflect the low sample size used (*N* = 1 for the Long-Storage group). Other possibilities exist independently of a low N which should be addressed. We do not believe the finding was related to time in storage as previous studies have observed that the length of time in cryopreservation had minimal effects on cells after reconstitution (Aird et al., 1992; Yuan et al., 2016; Miyamoto et al., 2018; Mun et al., 2019; Prinelli et al., 2021; Li et al., 2022; Gokarn et al., 2023; Xue et al., 2024). Also, we have shown that SOSR-COs stored for 6 months before reconstitution displayed no difference in developmental trajectory compared to controls after rewarming and culture to day 90 p.d. (Figure 7). The most likely cause for the difference between controls and the long-Vitri condition is batch-to-batch variation. The controls and short-Vitri conditions were derived at a separate time from the long-Vitri condition. However, we cannot rule out toxic or anti-oxidant effects of some CPAs, including DMSO which was used in our protocol (Best, 2015), or effects of the cryopreservation process in general, as recently described hCO cryopreservation methods showed an increase in the progenitor cell zone with a slight but non-significant increase in SOX2^+^ cells (Ramani et al., 2024; Xue et al., 2024).

Another possibility is that the changes reflect the different timing of hCO vitrification. For long-term storage, hCOs were vitrified at day 35 p.d. instead of day 20 p.d., and the short-Vitri condition also presented differences in CTIP2^+^ and Casp3^+^ cell numbers after day 35 p.d. vitrification. hCOs at 35 days p.d. contain more heterogeneous and possibly more fragile cell populations. Previous work has shown that different cell types have variable tolerances to CPA concentrations and osmotic pressure (Fahy et al., 2004; Whaley et al., 2021). We believe that CPA penetration is unlikely to be the cause of these deficits as the day 20 MR-hCOs were much larger at the time of vitrification than day 35 SOSR-COs used in the long-term storage experiments, indicating that CPA penetration was not problematic. In addition to testing the timing of vitrification and assessing the potential consequences of vitrification on hCO development, more work is needed, including a larger sample size for the long-Vitri condition, to confirm a deficit in development and to optimize the protocol to address this potential issue.

The ability to minimize batch-to-batch variation between labs is important for maintaining high rigor in hCO research. To this end, we have found that hCO vitrification is a viable method to achieve high replicability. Organoids sent across the country after 6 months of storage show similar levels of all markers tested except for MKI67. The difference in MKI67 and the trends toward differences in Casp3 and SOX2 may indicate negative impact on the progenitor pool. However, this did not seem to impact the differentiated cell types since we do not see any difference in mature cell type markers in the SOSR-COs or MR-hCOs at the same timepoint (Figures 3, 6). Since Ki67 was also reduced in the local-Vitri condition, the cause of these differences is likely due to a low sample number as this cell line at the same timepoint when used in previous experiments did not show this discrepancy. Another protocol for shipping hCOs long distance was recently published (Smith et al., 2025). The authors shipped individual 6- and 9-month-old MR-hCOs in 1.5 mL tubes on ice for overnight delivery. We believe our protocol offers advantages in comparison to this protocol. First, complications in shipping can occur, including delays in shipping or misplaced packages. The LN_2_ dewar in our protocol can hold temperature for several days before temperature fluctuations occur, something that an icepack in a polystyrene container cannot achieve. Moreover, because the hCOs are already vitrified there is no risk of metabolite buildup or depletion of nutrients in the case of live hCO shipping.

Two recently published articles describe slow-cooling methods using a novel freezing media cocktail (MEDY) and a commercially available media (CS10, Stemcell Tech) that were able to cryopreserve cortical brain organoids (Ramani et al., 2024; Xue et al., 2024). This work shows that neural tissues that have undergone a freeze/thaw can maintain their organization and function with minimal impact on cell type diversity and excitability. In comparison to Xue et al., our method offers the advantage of significantly reduced time to cryopreservation and does not need to be augmented based on organoid size. Our method also does not require special culture conditions post thawing. Finally, the Cryolock^®^ can hold up to 6 organoids which reduces the footprint in the LN_2_ tank. Ramani et al. presented less characterization of rewarmed organoids, but the authors do show differences in size and growth which is not observed using our method. They also found that their day 35 frozen organoids had poorer viability than their day 20 freezing timepoint. In contrast, although our organoids vitrified at 35 days p.d. show significant differences in several cell types, we did not note any issues with organoid viability. Both of the other published hCO cryopreservation methods yielded an increase in the number of SOX2^+^ cells within the progenitor zone which was not observed with our method. In general vitrification prevents ice formation, and the 1-min rewarming step limits the potential shearing damage that may occur due to ice shifting which frequently occurs with slow freezing protocols (Mazur, 1984), which may be the reason for our high viability and cell diversity maintenance.

In summary, we demonstrate that vitrification is a viable alternative strategy for the cryopreservation of hCOs using two organoid derivation protocols. We have also shown that it is possible to store vitrified hCOs for over a year and ship them long-distance with minimal alterations in organoid growth and cellular diversity. Because we only tested one CPA cocktail formulation and only performed vitrification at two timepoints (days 20 and 35 p.d.), the protocol may be further optimized by exploring other time points or CPA cocktails. In this study, we used LN_2_ because we had previous experience with this method, the Cryolock^®^ is optimized for LN_2_ and it is the most readily available cryogen in our laboratory. However, other cryogens exist including liquid ethane or propane which yield higher cooling rates than LN_2_ and could be a better fit for the cryopreservation of hCOs. Comparisons between cryogens for effectiveness in preserving hCOs will be needed. In the future we should also examine the exact internal cooling rate of the hCOs at different sizes to ensure optimal cryopreservation. Our protocol is also limited in scalability due to the size of the Cryolock^®^, although, we are working with the manufacturer to adapt the technology to allow for more organoids to be stored in the same Cryolock^®^. Nonetheless, the vitrification method will be useful for sharing hCOs with labs that are interested in adding human organoid models for their research but do not have the necessary expertise with hPSC culture methods or cannot afford the costs of hCO generation. Additionally, this protocol may allow for the creation of brain organoid biobanks to avoid sample loss and to allow for large-scale sharing of patient tissue.

## Data Availability

The original contributions presented in the study are included in the article/Supplementary material, further inquiries can be directed to the corresponding author.

## Supplementary material

Supplementary Figure 1 Confirmation of pluripotency markers in reprogrammed iPSC lines. **(A)** 79b iPSCs derived from human foreskin fibroblast cells were stained for **(Ai)** NANOG (green) **(Aii)** OCT4 (green) and **(Aiii)** SOX2 (green). All counterstained with Hoechst (blue). Scale bar: 200 μm.

Supplementary Figure 2 Vitrification does not impact cell growth or death shortly after reconstitution. Organoids at 2 days p.v. were immunostained for and Casp3 to examine proliferation and cell death. **(A)** Confocal images of MKI67+ (gray) and Casp3+ (red) immunolabeling in control (left) and post-vitrified (right) SOSR-COs. **(B,C)** The amount of immunolabeling for MKI67 **(B)** and Casp3 **(C)** was not statistically different between groups (*p* = 0.96, *p* = 0.12, respectively). Statistics = unpaired *t*-test. Scale bar = 500 μm (large), 50 μm (inset). *N* = 2 experiments 4–5 hCOs/line/batch.

Supplementary Video 1 Steps to perform vitrification of cortical hCOs.

Supplementary Video 2 Steps to warm hCOs after vitrification.
