# Supplementary material for: Cryopreservation of human cortical organoids using vitrification
Source: Front Mol Neurosci. 2026 Sep 8;19:1913908. doi: 10.3389/fnmol.2026.1913908 (PMC13595579; doi:10.3389/fnmol.2026.1913908)
Supplement: Supplementary file 1 [file Data_Sheet_1.pdf]

Supplemental Experimental procedures

Vitrification

On day 20 p.d., organoids (hCOs) were randomly split into two groups: controls (unvitrified) and vitrified (Vitri) hCOs, with the latter undergoing vitrification and subsequent rewarming. The cryoprotectants used in this protocol are toxic to cells and therefore the protocol should be performed with some haste to avoid damaging the hCOs. A graphical representation of this protocol can be found in Figure 1 and live instructions in Supplemental Videos 1 and 2.

Note: CPAs are toxic to cells. Researchers should work quickly to avoid damaging the organoids.

<u>Preparation</u>: Place one 70µL droplet of Washing Solution (WS) on the lid of a 4-well plate (Fisher, #14444). Place two 70µL droplets of Equilibration Solution (ES) to the right of the WS droplet with narrow but equal spacing. Repeat this for every 3-6 organoids. Place 500µL of ES in the top two wells of a 4 well-plate and place 500µL of Vitrification Solution (VS) in the bottom two wells. Each well can be used for 2 rounds of vitrification (6-12 organoids). In a HERAguard ECO clean bench, (Thermo) or similar bench, place a stereoscope, a Stripper® CC micropipetter with 600µm tip (Cooper Surgical, #MXL3-STR-CC; #MXL3-600), Cryolocks® (IVFStore, #SCL-R-CT-B) in a 7×6×4.5 polystyrene container filled with liquid nitrogen (LN_2) containing aluminum Cryo-Canes (Thermo, #5015-0002) affixed with a 13 mm goblet (IVF store, #13-100-CLR), 8-inch forceps (Fisher, #3020AFMT), the 4 well plates, lids containing vitrification solutions and a p200 pipette with cut off pipette tips. 24-well plates (Fisher, #09-761-146) and lids can also be used instead of 4-well plates if desired. The number of hCOs used per round (3–6 organoids) was dependent on organoid size and the number that can fit on the Cryolock® tip.

Note: when transferring hCOs between solutions the researcher should only transfer the hCOs and avoid adding any liquid from the previous solution to the following one.

1) Inside a laminar flow hood, place organoids into a non-tissue culture-treated 35mm petri dish containing 2-3mL of their usual medium (protocol dependent) and move them to the clean bench. Place the remaining organoids back into the incubator.

2) Move 3-6 hCOs from the 35 mm dish using a cut-off p200 pipette and place them in the 70µL droplet of WS. Use the p200 tip to merge the WS and first ES droplets together. Incubate for 3 min at RT.

3) Repeat step 2 by dragging the second droplet of ES into the previously merged droplets. Incubating for 3 min at RT.

4) Remove the hCOs from the droplet and expel them in the well containing 100% ES and incubate for 10 min at RT.

5) Quickly move the hCOs from ES to one well containing VS and incubate for no more than 1 min at RT.

Note: hCOs will float due to the high sucrose concentration. Repeatedly, but gently, pipette organoids below the surface so they remain submerged and can equilibrate to the medium.

6) Quickly move the hCOs to the beveled edge of the Cryolock®.

Note: Instructions on Cryolock® use can be found here.

7) Use the Stripper tool to remove all excess VS from the Cryolock® and evenly distribute hCOs along the length of the Cryolock®. Avoid placing them near the black line at the tip or the base. Removal of VS from the Cryolock® allows for minimum volume cooling (MVC) and prevents ice crystal formation[1].

   Note: Based on our experience. leaving VS on the Cryolock® resulted in organoid dissociation and loss of the organoid upon rewarming.

8) Once VS is removed and hCOs are arranged, plunge the tip of the Cryolock® directly into the LN_2.

9) Using the 8-inch forceps affix the cap of the Cryolock® securely to the base while the Cryolock® tip is still submerged in the LN_2.

10) Quickly move the Cryolock® into the goblet submerged in LN_2 to minimize the time the tip of the Cryolock® is out of the LN_2.

11) Once all hCOs have been vitrified, move the goblet containing the Cryolocks® into a cryo-storage dewar containing LN_2 for long-term storage.

Rewarming:

Preparation: Add 2mL of Rewarming Solution (RS) into a 35mm petri dish (and in a separate 35 mm dish add 2mL of organoid culture medium (this is a short-term storage) and place both in a 37°C incubator. Finally, prepare one or more dishes/plates containing the regular organoid medium for long-term culture and place at 37°C for 30 min. During this incubation, in an ECO clean bench, set the heated stage stereoscope to 37°C. Next to the stereoscope, place a polystyrene box containing the cane with Cryrolocks® inside submerged in LN_2. Inside a biosafety cabinet, add 500µL Diluent Solution (DS) to 1 well of a 4-well plate and 500µL of WS to two wells of the plate (this is good for 2 rounds of vitrification with 3-6 organoids each). Place the 4-well plate, a pre-cut p200 pipette tip, and 8-inch forceps in the ECO clean bench.

Note: Transfer as little of the previous solution into the next step to avoid CPA toxicity.

12) Remove the RS-containing 35mm dish from the 37°C incubator and place it on the heated stage of the stereoscope and remove the lid (the RS must be maintained at 37°C for proper warming). *If the user does not have access to a heated stage stereoscope, then they should prepare multiple plates containing RS and cycle them in the incubator, so each rewarming is at 37°C.*

13) Grab the end of a Cryolock® and keep the capped end submerged in the LN_2. Using the forceps, gently remove the cap of the Cryolock® and place it aside.

14) Quickly remove the Cryolock® from the LN_2 and fully submerge the tip into the petri dish containing RS for 1 min (RS must be at 37°C or the hCOs will dissociate).

15) Move the hCOs to the well of the 4-well plate containing DS and incubate for 3 min. During the following three incubations, place the RS-containing petri dish back in the incubator until the next round of rewarming.

16) Move hCOs from DS to the first well of WS and incubate for 5min. Repeat this step in the second well of WS.

17) Remove the 35mm dish containing the hCO medium, transfer the hCOs into this dish and place back into a 37°C incubator, 5% CO_2.

18) Repeat the above steps until all organoids have been rewarmed.

19) Remove the 35mm dish containing hCOs and transfer the organoids to the long-term storage vessel(s) prepared earlier using a cut-off p200 tip and culture as usual.

Cryolock® Maintenance

Used Cryolocks® can be gently wiped with sterile water and allowed to air dry in a biosafety cabinet. These can be reused 2-3 times before they become too fragile for reuse.

Supplemental Table 1

**Reagent**	**Manufacturer**	**Cat. #**	**Concentration**<br>**Based on a 10mL volume**	
**Washing Solution (WS)**				
Knockout Serum Replacement (KOSR)	Invitrogen	#10828010	20%	2 mL
DMEM F/12 (15 mM HEPES)	Invitrogen	#11320032	80%	8 mL
**Equilibration Solution (ES)**				
Ethylene Glycol	Millipore-Sigma	#109621	7.5 %	0.75 mL
DMSO	Sigma	#D2650-100mL	7.5%	0.75 mL
KOSR	Invitrogen	#10828010	20%	2 mL

DMEM F/12 (15 mM HEPES)	Invitrogen	#11320032	65%	6.5 mL
**Vitrification Solution (VS)**				
Ethylene Glycol	Millipore-Sigma	#109621	15%	1.5 mL
DMSO	Sigma	#D2650-100mL	15%	1.5 mL
KOSR	Invitrogen	#10828010	20%	2 mL
Sucrose	Fisher	#AAJ6514836	0.5 M	
DMEM F/12 (15 mM HEPES)	Invitrogen	#11320032		Fill to 10 mL
**Rewarming Solution (RS)**				
KOSR	Invitrogen	#10828010	20%	2 mL
Sucrose	Fisher	#AAJ6514836	1 M	
DMEM F/12 (15 mM HEPES)	Invitrogen	#11320032	Fill to 10 mL	Fill to 10 mL
**Diluent Solution (DS)**				
KOSR	Invitrogen	#10828010	20%	2 mL
Sucrose	Fisher	#AAJ6514836	0.5 M	
DMEM F/12 (15 mM HEPES)	Invitrogen	#11320032	Fill to 10 mL	Fill to 10 mL

1. Hochi, S. (2022). Cryodevices developed for minimum volume cooling vitrification of bovine oocytes. Anim Sci J *93*, e13683. 10.1111/asj.13683.
